# Pharmacologic Interventions for Fracture Risk Reduction in the Oldest Old: What Is the Evidence?

**DOI:** 10.1002/jbm4.10538

**Published:** 2021-09-05

**Authors:** Jad G Sfeir, Robert J Pignolo

**Affiliations:** ^1^ Robert and Arlene Kogod Center on Aging Mayo Clinic Rochester MN USA; ^2^ Division of Endocrinology Diabetes, Metabolism, and Nutrition, Mayo Clinic Rochester MN USA; ^3^ Division of Geriatric Medicine and Gerontology Mayo Clinic Rochester MN USA

**Keywords:** AGING, ANTIRESORPTIVES, FRACTURE PREVENTION, OSTEOPOROSIS, SARCOPENIA

## Abstract

With an increasingly older population, the proportion of patients 85 years or older seeking interventions to protect their musculoskeletal health is growing. Osteoporosis in the geriatric population presents unique diagnostic and therapeutic challenges. Multimorbidity, frailty, falls, polypharmacy, and other neurobehavioral factors influence our approach to fracture prevention in this population. The vast majority of the evidence from clinical trials establish pharmacologic fracture efficacy in postmenopausal women. The evidence is scarce for the oldest old men and women, a population also at risk for adverse events and mortality. Most studies show continued efficacy of pharmacologic interventions in this age group, although they are largely limited by small sample sizes. We herein review the available evidence of pharmacologic interventions for fracture risk reduction in this population and explore the emerging senotherapeutic interventions in the pipeline. © 2021 The Authors. *JBMR Plus* published by Wiley Periodicals LLC on behalf of American Society for Bone and Mineral Research.

## Introduction

After bone accrual to reach a mature skeleton and a peak bone mass by the third or fourth decade of life, age‐related bone loss occurs at a rate of 0.5% to 1.5% per year in both sexes.^(^ ^1^, ^2^ ^)^ Microarchitectural data show that cortical bone is lost starting around menopause in women but not until the seventh or eighth decade in men. Trabecular bone, on the other hand, is lost beginning in young adulthood in both men and women and continues throughout life, with an acceleration occurring around menopause in women.^(^ ^3^, ^4^ ^)^ The pathophysiology of age‐related bone loss is complex and not completely understood. There is reduction in the rate of bone formation, likely due to progressively limited function and/or number of osteoblasts, as well as an increase in the number of osteoclastic units.^(^ ^5^ ^)^ It is estimated that a loss of up to 50% of bone mass is achieved by age 80 years. The loss of skeletal mass translates into an increased risk of low‐trauma fragility fractures, which are the hallmark of osteoporosis.

Muscle mass is also similarly subject to age‐associated decline, at a rate of 1% to 2% per year after age 50 years, with men having a tendency for more muscle mass loss with age. After age 70 years, there is a loss of about 25% to 40% per decade of muscle strength.^(^ ^6^, ^7^, ^8^ ^)^ The increasingly recognized cross‐talk between bone and muscle has led to the use of the term osteosarcopenia to describe the age‐related musculoskeletal decline. The wide heterogeneity in clinical definition of sarcopenia and lack of highly reproducible measurement tools contribute to the underrecognition of osteosarcopenia in the clinic. Consequently, its prevalence has varied in the literature, ranging between 5% and 40%, with a higher prevalence among adults with falls and fractures.^(^ ^9^, ^10^ ^)^

Both peak bone mass and peak muscle mass are generally higher in men than in women, and thus the age‐related decline does not translate into an equal clinical risk for a given age among both sexes.

With an increasingly older population, a larger proportion of patients in the oldest old age group (85 years or older) are seeking intervention to reduce fracture risk and maintain a precious independence in their mobility.

In addition to the complexity of pathophysiologic interplay of musculoskeletal health in this population, additional factors influence our approach to osteoporosis in the elderly, such as multimorbidity, polypharmacy, type of dwelling, and physical and cognitive dysfunction. Recent data have also suggested a negative association between increased sympathetic outflow in older adults and bone microarchitecture. Commonly used fracture risk assessment tools (eg, FRAX calculator) do not fully account for frailty or sarcopenia and may thus underestimate the fracture risk in older adults.^(^ ^11^ ^)^ The Fracture Risk Assessment in Long‐term Care (FRAiL) calculator is a recently developed tool that relies on a host of clinical factors, including physical performance and muscle function, to predict the 2‐year risk for hip fractures in adults residing in nursing homes.^(^ ^12^ ^)^

The management of osteoporosis and/or osteosarcopenia in older adults is thus significantly more intricate than that of younger patients. In addition to addressing underlying chronic illnesses that may be contributing to frailty and skeletal fragility, our “osteoporosis prescription” should include intervention to reduce risk of future falls and fractures while maintaining, or even improving, overall musculoskeletal health. It is thus important to understand the evidence behind currently available pharmacotherapeutics to generate a pragmatic and personalized management plan.

In this review, we will examine the evidence of current fracture risk reduction pharmacologic interventions in the oldest old. Where available, we have relied on clinical trials that exclusively examined the interventions in women and men aged 85 years or older. In a number of instances, such studies were not available and thus the evidence was inferred from studies in population 70 to 75 years or older. In addition, we have included the reported estimates of relative risk reduction from these studies. As highlighted by Seeman and colleagues,^(^ ^13^ ^)^ the use of absolute risk reduction and number needed to treat would better showcase the importance of pharmacologic intervention for individuals in this age group given their higher baseline absolute risk.

## Nonpharmacologic Interventions

### Physical activity

A major component of the “osteoporosis prescription” in the oldest old is increased physical activity. Multiple forms of exercise have been subject to investigation in older adults, with clear evidence that exercise reduces the risk of falls and may modestly improve balance and bone mineral density (BMD).^(^ ^14^, ^15^, ^16^ ^)^

The beneficial impact seems to be closer to the time of intervention, highlighting the importance of individualizing the exercise prescription in order to achieve a sustainable and reproducible home program.

### Calcium and vitamin D

There is evidence to suggest that men and women older than 65 years with low serum 25‐hydroxyvitamin D (25(OH)D) concentrations (<10 ng/mL) are at greater risk for decreased muscle strength and increased hip fractures rates.^(^ ^17^, ^18^ ^)^ Vitamin D supplementation may improve BMD and muscle function, particularly in patients with hypovitaminosis D.^(^ ^19^ ^)^ The effect of vitamin D on risk of falls remains controversial but may be of particular importance in institutionalized older adults.^(^ ^20^, ^21^, ^22^ ^)^

Vitamin D deficiency, defined as 25(OH)D levels of less than 20 ng/mL, is noted in 20% to 100% of community‐dwelling older adults.^(^ ^23^ ^)^ In a study looking at 125 consecutive patients aged 75 or older admitted to a geriatrics unit, vitamin D deficiency, defined as 25(OH)D levels of less than 30 ng/mL, was prevalent in 85% of subjects; about half had levels below 10 ng/mL.^(^ ^24^ ^)^ The same pattern was observed in the oldest old subgroup.

Institutionalized elderly tend to have lower serum 25(OH)D levels than those living in the community.^(^ ^25^ ^)^ Low 25(OH)D levels are associated with poor musculoskeletal outcomes, including vertebral and hip fractures.^(^ ^26^ ^)^ Results from the English Longitudinal Study of Aging (ELSA) showed significant correlation of 25(OH)D less than 12 ng/mL with poor muscle strength and performance as well as low grip strength in the community‐dwelling adults 80 years or older.^(^ ^27^, ^28^ ^)^ The increased prevalence of vitamin D deficiency with age is multifactorial, in part due to decreased sun exposure and reduced efficiency of the production pathway but also decreased adequate supplementation of vitamin D.^(^ ^25^ ^)^

Although adequate vitamin D supplementation restores serum 25(OH)D levels, the evidence for clinically meaningful outcomes is limited. In a randomized control trial (RCT) of ambulatory healthy older women residing in nursing homes in France (mean age 84 years and mean 25(OH)D of 16 ng/mL), 800 IU of vitamin D and 1200 mg of elemental calcium were associated with a significant reduction in hip (odds ratio [OR] = 0.7; 95% confidence interval [CI] 0.53–0.97) and nonvertebral fractures compared with placebo.^(^ ^29^ ^)^ An RCT of older men and women with history of falls (mean age 78 years and mean 25(OH)D of 18 ng/mL), various doses of vitamin D failed to reduce risk of falls, although all groups had improvement in overall physical performance.^(^ ^30^ ^)^ A pooled analysis of RCTs looking at dose of vitamin D supplementation and osteoporotic fractures showed a significant risk reduction of both hip (relative risk [RR] = 0.7, 95% CI 0.58–0.86) and nonvertebral fractures (RR = 0.86, 95% CI 0.76–0.96) in older adults (65 years or older) independent from the type of dwelling.^(^ ^31^ ^)^ The prespecified analysis of those aged 85 or older (345 in the intervention arm and 1985 controls) was, however, not statistically significant (hip fracture RR = 0.54, 95% CI 0.25–1.20 and nonvertebral fracture RR = 0.87, 95% CI 0.59–1.30), in part owing to reduced power and sample size in this subgroup.

The National Academy of Medicine (formerly the Institute of Medicine) recommends 1200 mg of calcium per day and 800 IU of vitamin D per day in all adults aged 70 years or older. A higher vitamin D dose may be needed in those with established osteoporosis and/or sarcopenia. A total daily calcium intake not exceeding 2000 to 2500 mg is considered safe by the National Osteoporosis Foundation and the American Society for Preventative Cardiology. Vitamin D doses up to 2000 IU per day are similarly very safe.^(^ ^22^ ^)^

## Pharmacologic Interventions

### Hormone therapy

Sex steroids are very potent antiresorptives and play a central role in skeletal health. The use of estrogen in postmenopausal women and testosterone in men to maintain musculoskeletal health has received considerable share in the literature. In the oldest old age group, the potential benefits from these interventions have been overshadowed by concerns of safety.

Despite strong evidence of fracture reduction with estrogen in postmenopausal women, the increased risk for cardiovascular events and breast cancer observed in the Women Health Initiative trials have significantly limited their use beyond the first decade after menopause.^(^ ^32^ ^)^

An initial RCT of testosterone scrotal patch in older men (mean age 73 years) with low‐normal testosterone (mean 367 ng/dL) showed similar increases in spine BMD at 3 years compared with placebo and no change in hip BMD or muscle strength.^(^ ^33^, ^34^ ^)^

Another interventional trial of men (mean age 71 years) with mildly low testosterone (mean 288 ng/dL) using intramuscular testosterone showed significant increase in both spine and hip BMD (mean increase of 10.2% and 9.3%, respectively) compared with placebo (1.3% and −0.2%, respectively) at 3 years.^(^ ^35^ ^)^ Physical performance and handgrip strength also showed significant increases with testosterone treatment. To note, however, the testosterone dose used in this trial was supraphysiologic.

Similarly, high‐dose testosterone gel treatment for 6 months showed significant improvement in muscle strength and some modest improvements in physical function in an RCT of elderly men (mean age 74 years) with mildly low testosterone levels (mean 248 ng/mL).^(^ ^36^ ^)^

In the Testosterone Trials, older men (mean age 72 years) with moderately low baseline testosterone levels (mean 234 ng/dL) were randomized to receive either placebo or transdermal testosterone gel with dose adjustments to reach levels within the normal range for young men.^(^ ^37^ ^)^ Although no significant improvement in the 6‐minute walk test was noted in the Physical Function Trial (*n* = 387), an increase in the walking distance was found when participants across all trials were included (OR = 1.76, 95% CI 1.21–2.57).^(^ ^37^ ^)^

In the Bone Trial (*n* = 211), there was an increase in spine and hip volumetric BMD by quantitative computed tomography (QCT; mean of 4.2% and 1.3% greater increase from baseline compared with placebo, respectively), with the greatest increase found in the trabecular compartments. There was also a greater increase in bone strength by finite element analysis (FEA) with testosterone treatment at both sites. Areal BMD had a modestly greater increase at the lumbar spine in the testosterone arm, but no differences were noted at the hip.^(^ ^38^ ^)^

In frail older men (mean age 74 years) with low‐normal testosterone, testosterone gel treatment for 6 months improved isometric muscle strength but not physical performance.^(^ ^39^ ^)^

Testosterone treatment in older men is associated with increased risk for cardiovascular adverse events (OR = 5.8, 95% CI 2.0–16.8), including acute coronary events, stroke, hypertension, and peripheral edema.^(^ ^40^ ^)^

Only 138 men in the Testosterone Trials underwent CT angiography, which showed an increase in noncalcified coronary artery plaque volume with testosterone treatment. Participants in the testosterone arm across all trials (*n* = 394) had no increase in cardiovascular events after 1 year of treatment, but a higher proportion had increase in PSA by ≥1.0 ng/mL and a hemoglobin ≥17.5 g/dL compared with the placebo arm.^(^ ^37^ ^)^

### Alendronate

A post hoc analysis of older women enrolled in the Fracture Intervention Trial (FIT) trial showed similar efficacy for fracture risk reduction with alendronate in women aged 75 years or older when compared with those <75 years (number needed to treat 8 and 9, respectively, to prevent 1 vertebral fracture and 15 and 13, respectively, to prevent 1 clinical fracture).^(^ ^41^ ^)^ The original trial, however, only included women up to the age of 82 years.^(^ ^42^ ^)^

In a retrospective case–control study using the national Swedish Fractures and Fall Injuries in the Elderly Cohort (FRAILCO) of men and women aged 80 years or older (mean age 85.7 years) and having a prior history of fracture, 1961 subjects receiving alendronate for a mean of 3.5 years were compared with 7844 untreated controls.^(^ ^43^ ^)^ During a mean follow‐up of 18.1 months, incidence of hip fractures (hazard ratio [HR] = 0.62, 95% CI 0.49–0.79), major osteoporotic fractures (HR = 0.68, 95% CI 0.56–0.83), and any fractures (HR = 0.78, 95% CI 0.67–0.90) were all reduced in the alendronate group and maintained statistical significance after multivariate adjustments. Interestingly, alendronate was also associated with a reduction in all‐cause mortality (HR = 0.88, 95% CI 0.82–0.95) even after adjustment for comorbidities.

The FRAILCO data showed a significantly higher prevalence of mild upper gastrointestinal (GI) symptoms with alendronate (2.3% versus 1.4% in nontreated controls, *p* < 0.01) but no difference in peptic ulcers (1.2% versus 1% in nontreated controls, *p* = 0.62).

Despite the lack of a direct measure of the absolute fracture risk reduction in the oldest old, these results show continued efficacy of alendronate in reducing fragility fractures in older age. The mortality benefit needs to be explored further, particularly with emerging mortality data with zoledronic acid (discussed later).

### Risedronate

The Hip Intervention Program (HIP) randomized trial included an arm where women 80 years of age or older with at least one non‐skeletal risk factor for hip fracture (difficulty standing from a sitting position, a poor tandem gait, a fall‐related injury during the previous year, a psychomotor score of 5 or less on the Clifton Modified Gibson Spiral Maze test, current smoking or smoking during the previous 5 years) were randomized to receive daily 2.5 mg of risedronate (*n* = 1281), 5 mg of risedronate (*n* = 1292), or placebo (*n* = 1313) for 3 years.^(^ ^44^ ^)^ About 45% of these women had a prevalent vertebral fracture, and 45% had serum 25(OH)D <16 ng/mL. Risedronate (combined data of both doses) showed a nonsignificant reduction in incident morphometric hip fractures (RR = 0.8, 95% CI 0.6–1.2). Notably, most of the women in this arm had an unknown baseline bone density, and only 16% had evidence of low BMD.

A subsequent post hoc analysis of the HIP trial data included women aged 70 to 100 years with established osteoporosis (by BMD criteria and presence of at least one vertebral fracture) and showed a reduction in incident hip fractures (RR = 0.54, 95% CI 0.32–0.91).^(^ ^45^ ^)^

A pooled analysis from the HIP trials as well as the Vertebral Efficacy with Risedronate Therapy‐Multinational (VERT‐MN) and VERT‐North America (NA) trials reviewed data from women 80 years or older (mean age 83 years) with osteoporosis (by BMD criteria, mean *T*‐score −3.05) receiving 5 mg risedronate or placebo daily.^(^ ^46^ ^)^ Most women (84%) had a prevalent vertebral fracture at baseline. Risedronate was associated with an 81% reduction in the incidence of vertebral fractures at 1 year (HR = 0.19, 95% CI 0.09–0.40) and 44% at 3 years (HR = 0.56, 95% CI 0.39–0.81). There was no significant difference in the incidence of nonvertebral fractures.

In the HIP data as well as the pooled data, no differences in adverse drug events (ADEs) were noted in either risedronate doses compared with placebo, including serious ADEs and upper GI symptoms.^(^ ^44^, ^46^ ^)^ Interestingly, 20% to 30% of subjects in both treatment and placebo groups experienced an upper GI symptom.

The initial HIP analysis included women at risk for osteoporosis, whereas subsequent analyses focused on those women with established osteoporosis using BMD and/or fracture criteria. As such, there is sufficient evidence for the efficacy of risedronate to reduce vertebral fracture risk in the oldest old with osteoporosis. The evidence for nonvertebral fracture reduction, however, remains unclear and largely inferred from data in the 70 to 79 years age group, given lack of power in the older group.

### Zoledronic acid

A post hoc analysis of the pooled data from the Health Outcome and Reduced Incidence with Zoledronic Acid One Yearly (HORIZON) Pivotal Fracture Trial and the HORIZON Recurrent Fracture Trial included women 75 years or older with a history of osteoporosis (by BMD or fracture criteria) receiving 5 mg of zoledronic acid (*n* = 1961, mean age 79.3 years) or placebo (*n* = 1926, mean age 79.6 years) annually for 3 years.^(^ ^47^ ^)^ The HORIZON Recurrent Fracture Trial included 292 women aged 85 years or older (140 in the zoledronic acid group and 152 in placebo) randomized after a recent hip fracture.^(^ ^48^ ^)^ There was a reduction in the risk of clinical vertebral (HR = 0.34, 95% CI 0.21–0.55), nonvertebral (HR = 0.73, 95% CI 0.6–0.9), and any fractures (HR = 0.65, 95% CI 0.54–0.78) but not hip fractures (HR = 0.82, 95% CI 0.56–1.2) at 3 years. In addition, an increase of femoral neck (+5%) and total hip (+6.3%) BMD were noted.

Zoledronic acid efficacy has been suggested to extend beyond fracture risk reduction. In older women with osteopenia (mean age 71 years), zoledronic acid infusion every 18 months resulted in a significant reduction in total cancer incidence over 6 years (RR = 0.69, 95% CI 0.53–0.90), which was most notable for a reduction in breast cancer incidence (RR = 0.59, 95% CI 0.35–0.98).^(^ ^49^, ^50^ ^)^ Although there was no significant overall survival benefit, a reduction in mortality in the 1688 women without an incident fragility fracture was noted (HR = 0.51, 95% CI 0.30–0.87). The HORIZON Recurrent Fracture Trial showed a 3.3% absolute risk reduction in mortality (HR = 0.72, 95% CI 0.56–0.93) in the overall cohort (mean age 74.4 years), which was, however, not found in the pooled analysis of women 75 years and older in the HORIZON trials.^(^ ^47^, ^48^ ^)^ The Zoledronic Acid in Frail Elders to Strengthen Bone (ZEST) trial, albeit not powered for secondary functional outcomes, failed to show significant effect of a single infusion of zoledronic acid on functional status in frail women 65 years or older (mean age 85.5 years) residing in nursing homes or assisted living facilities.^(^ ^51^ ^)^ There was, however, a significant increase in BMD (3.9 ± 0.7% and 2.7 ± 1.0% higher than placebo at the lumbar spine and femoral neck, respectively); fracture data remain unpublished (NCT02589600).

Preclinical studies have suggested that part of the extra‐skeletal effects of zoledronic acid may be due to its activity on the senescence pathway (see Senolytics section below), but confirmatory studies are needed to elucidate better its role in influencing cellular senescence.^(^ ^52^ ^)^

The pooled HORIZON data showed a significant increase in post‐infusion inflammatory symptoms (pyrexia, myalgias, chills, bone pain, fatigue) in the zoledronic acid group (40% versus 20% in placebo) but no significant difference in serious ADEs.

Despite this lack of direct evidence of the fracture efficacy of zoledronic acid in the oldest old population, particularly in men, the vertebral and overall fracture risk reduction can conceivably be extrapolated from the HORIZON trials that included women in that age group.^(^ ^47^, ^48^, ^53^ ^)^ Interestingly, the data point toward possible non‐skeletal benefits of zoledronic acid, with potential mortality benefit that is independent from its fracture risk reduction. Further investigations into these outcomes are likely to emerge in the next few years.

### Denosumab

Subgroup analysis of women enrolled in the Fracture Reduction Evaluation of Denosumab in Osteoporosis Every 6 Months (FREEDOM) trial showed a reduction in vertebral (RR = 0.36, 95% CI 0.25–0.53) but not nonvertebral fractures (RR = 0.84, 95% CI 0.63–1.12) in women aged 75 years or older over 3 years.^(^ ^54^ ^)^ The trial included women with osteoporosis up to the age of 90 years.^(^ ^55^ ^)^ Additional post hoc analysis of these women (aged 75 to 90 years, mean age 78.2 years) showed a significant reduction of hip fracture incidence (absolute risk reduction 1.4%, *p* < 0.01).^(^ ^56^ ^)^

The post hoc analysis did not show significant increase in ADEs in women aged 75 years or older receiving denosumab, although a higher incidence of eczema and cellulitis were noted in the original FREEDOM trial.^(^ ^55^, ^56^ ^)^

Non‐skeletal effects of denosumab have received increased attention, particularly in patients with osteosarcopenia.

A proof‐of‐concept pilot study evaluated total body composition by dual‐energy X‐ray absorptiometry (DXA) and grip strength in 18 postmenopausal women (mean age 65 years) receiving denosumab and 20 women receiving either alendronate or zoledronic acid.^(^ ^57^ ^)^ At 3 years, significant increases in lean mass and grip strength were found only in the denosumab group.

When compared with zoledronic acid alone in 79 community‐dwelling older adults (mean age 80 years), denosumab showed higher improvements in balance measures and fear of falls, whereas the improvements in gait speed and timed up and go were comparable.^(^ ^58^ ^)^

In a pooled analysis of five RCTs that compared denosumab to placebo (mean age 71.8 years), a small but significant reduction in the rate of falls was found in both sexes (HR = 0.79; 95% CI 0.66–0.93); a greater reduction was found in those younger than 75 years of age.^(^ ^59^ ^)^

In a single‐site analysis of 38 elderly women (mean age 81, range 76 to 89 years) who participated in the FREEDOM trial, discontinuation of denosumab after 7 to 10 years of treatment resulted in significant decline in BMD at the lumbar spine (−8.1 ± 4.1%), femoral neck (−6.0 ± 4.7%), and total hip (−8.4 ± 4.6%).^(^ ^60^ ^)^ In addition, 5 of the 38 women sustained fragility fractures (four vertebral and one wrist) at least 1 year after treatment discontinuation.

Despite limited fracture efficacy data of denosumab in the oldest old age group, particularly for hip fracture, there is increasing evidence of its potential beneficial effects on falls, sarcopenia, and other non‐skeletal metabolic systems. The potential for superficial skin adverse reactions as well as the rapid bone loss and increased fracture risk that follow discontinuation of denosumab should be put in perspective when evaluating the benefit–risk ratio of denosumab in the oldest old.^(^ ^61^ ^)^

### Teriparatide (PTH 1‐34)

Subgroup analysis of women 75 years of age or older (mean age 78.2, range 75 to 86 years) who participated in the Fracture Prevention Trial (FPT) comparing teriparatide daily injections with placebo showed significant reduction in vertebral fracture incidence (RR = 0.35, *p* < 0.05) but nonsignificant reduction in nonvertebral fractures (RR = 0.75, p 0.661).^(^ ^62^ ^)^ In addition, BMD increased significantly at both the lumbar spine and femoral neck (+9% versus +2% in placebo, and +2% versus −0.5% in placebo, respectively). The trial included 23 women in the teriparatide group and 25 in the placebo group aged 80 years or older.

Fracture risk reduction was also found in the ≥75 years age group in the European Forsteo Observational Study (EFOS), in addition to self‐reported improvements in back pain, mobility, and activities of daily living.^(^ ^63^ ^)^

In a retrospective study looking at 316 men and women aged 80 years or older with osteoporosis (mean age 84.9, range 80 to 97 years) having received teriparatide prescription, about half (*n* = 154) discontinued the treatment due to lack of motivation (28%), relocation (18%), other illnesses (18%), death not related to teriparatide (17%), or adverse events (12%, most commonly nausea).^(^ ^64^ ^)^ A slightly lower rate of those <80 years of age discontinued the drug for similar reasons. In the ≥80 years age group, those who completed 2 years of therapy had a significant BMD increase by +14% at the lumbar spine and +4.5% at the femoral neck.

In older women participating in the FPT, diarrhea was notably increased in the teriparatide arm (10% versus 3% in placebo, *p* = 0.04).^(^ ^62^ ^)^ No serious ADEs were noted to be different from placebo.

Taken together, these results show that the benefits of teriparatide are insufficiently studied, to date, in the oldest old age group. Although potential BMD benefits can be extrapolated from the results in younger age groups, there are significant limitations to using teriparatide in older patients, not the least of which are practical ones related to the daily injection, which can result in poor compliance.

### Abaloparatide

Abaloparatide is a parathyroid hormone‐related protein (PTH‐rp) analog. The Abaloparatide Comparator Trial in Vertebral Endpoints (ACTIVE) trial included women up to the age of 86 years with osteoporosis comparing 18 months of abaloparatide with placebo.^(^ ^65^ ^)^ A subgroup analysis showed that those 75+ years of age had a nonsignificant reduction in nonvertebral fractures (RR = 0.29, 95% CI 0.08–1.07) but significant increases in BMD at the lumbar spine (least squares mean [LSM] +9.53%, 95% CI 8.05–11.01), total hip (LSM +3.54%, 95% CI 2.80–4.28), and femoral neck (LSM +3.81%, 95% CI 2.87–4.74).^(^ ^66^ ^)^

A post hoc analysis of those ≥80 years of age (*n* = 94, mean age 81.7 years) showed similar patterns of significant BMD increases (+12.1% at lumbar spine, +3.9% at total hip, and +3.6% at the femoral neck) and a nonsignificant decrease in fracture incidence.^(^ ^67^ ^)^ There were insufficient fracture events (1 nonvertebral fracture and 0 vertebral fracture in the abaloparatide group) to allow for adequate power for this analysis.

In the ACTIVExtend study, 56 of these ≥80‐year‐old women received 2 years of weekly oral alendronate after their original group assignment (abaloparatide versus placebo for 18 months).^(^ ^68^ ^)^ BMD increase continued at all sites in both groups but, similar to the ACTIVE study, there were too few fracture events (0 vertebral and 1 nonvertebral fracture in the abaloparatide‐to‐alendronate group) to allow for adequate analysis.

Overall ADEs in the ACTIVE trial were comparable between abaloparatide and placebo.^(^ ^65^ ^)^ In the ≥80‐year‐old group, however, 6 subjects discontinued the treatment (versus only 1 in placebo).^(^ ^67^ ^)^ This was higher than the discontinuation rate of the entire study cohort (24%). In addition, 3 deaths were noted in the treatment group (versus 0 in placebo). The deaths were due to ischemic heart disease, bronchiectasis, and sepsis. In the ACTIVExtend, no deaths were observed in either group, and the abaloparatide‐to‐alendronate group had slightly higher rates of musculoskeletal pain, sciatica, and dyspepsia.

As noted for teriparatide, efficacy data of abaloparatide in the oldest old is substantially limited by sample size and insufficient power. Considering the cost of the medication, need for daily injection, and other potential adverse events, abaloparatide use may be limited to select older adults at very high risk of fractures, particularly vertebral fractures.

### Romosozumab

Romosozumab, a sclerostin monoclonal antibody, is the newest osteoanabolic agent in the treatment of bone fragility. It is given as a monthly dose (two injections/dose) and, similar to denosumab, has to be administered by a health care provider.

The STRUCTURE phase 3 trial enrolled women up to age 90 (mean age 71 years) with osteoporosis and a history of fracture, having received at least 3 years of oral bisphosphonates, and were randomized to either romosozumab or teriparatide for 12 months.^(^ ^69^ ^)^ There was a significant increase in BMD at all three sites: lumbar spine (+9.8% versus +5.4% with teriparatide), total hip (+3% versus −0.5%), and femoral neck (+3.2% versus −0.2%). No age‐stratified data were provided.

Similar increases in BMD were found in the Active‐Controlled Fracture Study in Postmenopausal Women with Osteoporosis at High Risk (ARCH), which compared romosozumab for 12 months followed by alendronate for 12 months to alendronate alone for 24 months in women aged 55 to 90 years with osteoporosis.^(^ ^70^ ^)^ Enrollment was stratified by age (<75 and ≥75 years) and half of the population was aged 75 years or older. Romosozumab resulted in lower incidence of vertebral fractures (RR = 0.52, 95% CI 0.40–0.66), nonvertebral fractures (HR = 0.81, 95% CI 0.66–0.99), and hip fractures (HR = 0.62, 95% CI 0.42–0.92). The data were, however, presented in aggregate, and stratified results by age remain unpublished.

The same age stratification was done in the Fracture Study in Postmenopausal Women with Osteoporosis (FRAME), which compared romosozumab with placebo and included 30% of women in the ≥75 years age group (overall population mean age 70.9 years).^(^ ^71^ ^)^ There was a decrease in the incidence of new vertebral fractures in the romosozumab group (RR = 0.27, 95% CI 0.16–0.47) at 1 year but not in new nonvertebral fractures (HR = 0.75, 95% CI 0.53–1.05). The treatment effects were noted to be similar in the subgroup analysis (including the ≥75 years subgroup), although the data were not provided.

Romosozumab was also studied in men 55 to 90 years old with osteoporosis in the placebo‐controlled study evaluating the efficacy and safety of romosozumab in treating men with osteoporosis (BRIDGE) trial.^(^ ^72^ ^)^ Mean age was 72.4 years with about 40% of subjects aged 75 years or older. A subgroup analysis of those ≥70 years old showed significant increase in BMD at the lumbar spine (+10.2% difference from placebo), total hip (+2.8%), and femoral neck (+2.6%).

In women, the ARCH trial, but not the FRAME trial, showed an increase in adjudicated serious cardiovascular events with romosozumab. The difference could be attributed to a potential cardioprotective effect of alendronate, which was the comparator in the ARCH study. The BRIDGE trial showed an increase in adjudicated cardiovascular events in men in the romosozumab group (4.9% versus 2.5% in placebo). A meta‐analysis of cardiovascular events in patients with osteoporosis receiving romosozumab from six different trials showed nonsignificant risk of myocardial infarction (RR = 1.39, 95% CI 0.72–2.69), stroke (RR = 1.46, 95% CI 0.86–2.49), heart failure (RR = 1.26, 95% CI 0.66–2.42), and atrial fibrillation (RR = 1.12, 95% CI 0.49–2.54).^(^ ^73^ ^)^ The 4‐point major adverse cardiovascular events (MACE) composite was, however, slightly increased (RR = 1.39, 95% CI 1.01–1.90).

The experience with romosozumab remains relatively new when compared with other therapeutic options for osteoporosis. Although the clinical trials included men and women in the oldest old age group, the efficacy data are presented in aggregate and thus remain unclear. The cardiovascular risk associated with romosozumab certainly warrants further investigation in older patients, especially those at baseline increased risk for MACE.

### Agents in the pipeline

#### Senolytics

The future of pharmacotherapy for skeletal fragility and fracture risk reduction, particularly in older adults, is expected to follow a different approach of drug development. Advances in aging research have allowed for a large body of evidence demonstrating that cellular senescence is present at the site of development of chronic diseases, such as the bone microenvironment in patients with osteoporosis.^(^ ^74^, ^75^ ^)^ Cellular senescence is essentially an irreversible arrest of cell division accompanied by resistance to apoptosis (cell death). Activated at the cellular level after stressors such as DNA damage or oxidative stress, a number of mechanisms have been identified that confer a senescent state.^(^ ^74^, ^76^ ^)^ Senescent cells have been loosely described as “zombie cells” because of the fact that further cell proliferation is inhibited, without the possibility of reverting back into the cell cycle, but at the same time resisting cellular death.

With age, senescent cells accumulate in multiple tissues, particularly tissues central to the pathogenesis of chronic diseases. A cascade of events ensues that leads to tissue breakdown as well as local and systemic effects resulting in the development of age‐related chronic conditions and multimorbidity.^(^ ^74^ ^)^ The traditional medical model has been to address each of these diseases separately. The elucidation of senescence pathways that are common to multiple age‐onset conditions has allowed the development of a new approach, so called “translational geroscience,” in which targeting cellular senescence itself would allow for prevention of multiple chronic conditions, including osteoporosis.^(^ ^77^ ^)^

A phase 2 open‐label randomized clinical trial (NCT04313634) is currently making use of this new approach in women aged 70 years or older by targeting cellular senescence using senolytics, such as quercetin and dasatinib, to study the impact on bone remodeling and skeletal health.^(^ ^78^ ^)^

#### β‐adrenergic antagonists (β‐blockers)

A number of preclinical and clinical studies have suggested a negative relationship between sympathetic outflow and bone microarchitecture, particularly in postmenopausal women and older men.^(^ ^79^ ^)^ Observational studies have also highlighted a potential association between β‐blocker use and increase in BMD, as well as reduction in fracture rates in older adults.^(^ ^80^, ^81^ ^)^ Although the mechanisms remain unclear, there seems to be a stronger correlation with selective β_1_‐antagonists.^(^ ^82^ ^)^

Two ongoing clinical trials are evaluating the role of β‐blockers on prevention of bone loss (NCT04905277) and reduction in fracture rates (NCT04704947).

#### Vitamin K

Vitamin K plays multiple roles in bone metabolism. It inhibits osteoclastogenesis and stimulates osteoblastogenesis and is a co‐factor of a number of enzymatic activities. Clinical data on dietary vitamin K effects on BMD and fractures have been inconclusive.^(^ ^83^ ^)^ A meta‐analysis of small interventional studies had shown potential benefit on fracture risk reduction but not on BMD.^(^ ^84^ ^)^ More recently, a randomized trial of vitamin K2 versus placebo in postmenopausal women validated the prior data of lack of bone microarchitectural benefit at 3 years.^(^ ^85^ ^)^

#### Activin receptors

Activins belong to the superfamily of TGF‐β cytokines. They are abundant in skeletal muscles as well as the bone microenvironment, where they seem to regulate both osteoclastogenesis and osteoblast differentiation.^(^ ^86^ ^)^ Activin inhibition has been associated with increased bone mass and strength. Most of the studies to date, however, had small sample sizes and primarily evaluated safety and tolerability of activin receptor inhibitors. Efficacy data on functional outcomes have similarly been limited. Additional clinical trials, particularly in older adults, are needed to allow better assessment of their clinical use.

## Summary and Recommendations

Pharmacologic interventions for fracture prevention in the oldest old are largely driven by subgroup analysis of larger clinical trials and thus highly dependent on the recruitment efforts in this age group. The data presented herein indicate the substantial gap in evidence for fracture risk reduction with current pharmacotherapeutic interventions in men and women aged 85 years or older. Overall, bisphosphonates seem to have reasonable efficacy to be recommended as treatment in those with established osteoporosis (ie, at high or very high risk for fragility fractures). The risks associated with short‐term use do not seem to be significantly elevated in the oldest old. Patient education should focus on adequate administration of oral bisphosphonates to avoid gastric ulcers and on appropriate strategies to prevent post‐infusion inflammatory reaction with zoledronic acid.

Similarly, denosumab seems to have continued efficacy in older age with a limited side effect profile. It also provides a unique treatment option for patients with significantly reduced creatinine clearance. It is, however, a less attractive option given the major risk for rapid bone loss and incident vertebral fracture associated with a missed dose or discontinuation of use. In addition, denosumab may prove cumbersome for non‐ambulatory patients given the need for administration by ambulatory health care providers every 6 months.

Emerging data on beneficial non‐skeletal effects of zoledronic acid and denosumab are certainly noteworthy and offer a unique opportunity to target both skeletal muscle and bone in an older population at high risk for osteosarcopenia.

Long‐term risks of antiresorptive mediations include atypical femoral fractures and osteonecrosis of the jaw, both of which are associated with prolonged use of these drugs.^(^ ^87^ ^)^ This becomes increasingly important in institutionalized older adults in whom a regular review of the medication list and indications for continued use is essential. On the other hand, the risks for side effects associated with cumulative dosing of antiresorptive therapy may not be as important in those with lower life expectancy, resulting in a much more favorable risk‐to‐benefit balance in view of the high mortality after fragility fractures.^(^ ^88^, ^89^ ^)^

Both teriparatide and abaloparatide have very limited efficacy data in the oldest old based on inadequate power. Despite limited side effects, the cost and need for daily administration significantly limit their use in this population. It should be reserved for those at very high risk of recurrent fractures and should be accompanied by a solid compliance and follow‐up plan.

The evidence for romosozumab efficacy in the oldest old remains elusive and its cardiovascular risks concerning enough to recommend against its widespread use in this age group. Whether it will have future use in a subgroup of this population remains to be determined.

Finally, it is important to note that in a geriatric population, an individual patient's neurobehavioral and social environments should influence the choice of pharmacotherapy. A medication administered parenterally in a health care facility at regular intervals may be preferred over an oral weekly medication in patients with cognitive impairment but requires additional coordination for patient transportation and third‐party insurance coverage. On the other hand, a medication that can be safely interrupted may be a better option for patients with poor health care access or those with recurrent hospitalizations.

## Disclosures

The authors state that they have no conflicts of interest.

### Peer Review

The peer review history for this article is available at https://publons.com/publon/10.1002/jbm4.10538.
